# Intact but vulnerable: Affective working memory in social anxiety disorder

**DOI:** 10.1371/journal.pmen.0000506

**Published:** 2025-12-05

**Authors:** Arya Adyasha, Haroon R. Lone

**Affiliations:** 1 Department of Biological Sciences, Indian Institute of Science Education and Research Bhopal, Bhopal, India; 2 Department of Electrical Engineering and Computer Science, Indian Institute of Science Education and Research Bhopal, Bhopal, India; PLOS: Public Library of Science, UNITED KINGDOM OF GREAT BRITAIN AND NORTHERN IRELAND

## Abstract

Social anxiety disorder (SAD) is increasingly recognized as a disorder of cognitive-affective dysregulation, with growing evidence implicating affective working memory (AWM) as a core mechanism. This narrative review synthesizes findings from behavioral, electrophysiological, and neuroimaging studies to examine how socially and emotionally salient stimuli interfere with working memory (WM) processes in individuals with SAD. While general WM capacity often appears intact in neutral contexts, impairments emerge consistently when tasks involve socially threatening content or require executive control under cognitive load. Key disruptions include reduced flexibility, impaired updating, and sustained attentional capture by threat. These deficits are further moderated by individual symptom dimensions and task demands. The review critically evaluates current theoretical models and highlights methodological limitations in the field, proposing directions for future research. Clinical implications include the use of AWM-sensitive assessments and interventions that target executive regulation of emotion. AWM thus provides a mechanistic bridge linking cognition, emotion, and social dysfunction in SAD.

## 1 Introduction

A 2012 study found that the fear of public speaking ranks higher than the fear of death [1]—but for individuals with social anxiety disorder (SAD), this isn’t just anecdotal humor; it reflects a lived reality. SAD is more than shyness or introversion, according to standard definitions, it is a chronic and impairing condition marked by a persistent fear of scrutiny, humiliation, or rejection in social or performance situations [2]. Affecting an estimated 4.7-17% of the population over a lifetime [3], SAD undermines functioning across educational [4–6], professional [7], and interpersonal domains [8]. While always prevalent, its footprint has grown in the aftermath of the COVID-19 pandemic: prolonged isolation, social re-entry stress, and fatigue of digital communication have collectively sharpened social apprehension. A meta-analysis [9] reports a 25–30% surge in clinically relevant social anxiety symptoms post-pandemic, with young adults especially affected. Beyond its social manifestations, SAD has fundamentally shown compromised cognition [10–12]. Though often viewed as a disorder of social fear, decade long research suggests that its persistent impact may be best understood by examining the cognitive systems it disrupts [13], where attention [14], memory [15,16], and executive control [17,18] come together to shape a maladaptive self-perception [10,19].

Cognitive theories of SAD mentioned in meta reviews [10,20] emphasize biased information processing [21], including hyper-vigilance to threat [22], impaired attentional control [23], and a tendency to ruminate on socially threatening stimuli [24]. A growing body of literature suggests that these cognitive vulnerabilities could be underpinned by alterations in working memory (WM), a system responsible for the temporary storage and manipulation of information necessary for complex cognitive tasks [25,26]. Given the nature of the disorder and its known deficits in emotional and interpersonal information processing [9,27], affective working memory (AWM) is of particular relevance [28]. AWM refers to WM processes when emotionally salient content is involved [29]. Affective stimuli, particularly those involving social threat, pose a unique challenge to individuals with SAD by taxing limited WM resources and disrupting executive function [30–32].

Understanding the interplay between affective content and WM dynamics is essential, as deficits in AWM may not only maintain social anxiety symptoms but also impair an individual’s ability to regulate emotions in social contexts [30,33]. Yet despite a good number of studies on cognitive biases in SAD, the specific role of AWM remains under-theorized and fragmented across disparate paradigms. To the best of our knowledge, no prior review has integrated findings from behavioral performance, event-related potential (ERP) components, and neural correlates to characterize AWM functioning in SAD. Moreover, this digital era raises the urgency of examining how chronic affective load may exacerbate WM deficits in vulnerable populations.

This narrative review addresses a critical gap by synthesizing the current empirical literature on how affective and social threat stimuli modulate WM in individuals with SAD. By bringing together findings from experimental psychology, cognitive neuroscience, and affective science, we aim to (1) clarify the mechanisms linking social anxiety to impaired WM functioning, (2) highlight methodological inconsistencies that may account for mixed findings, and (3) propose directions for theory development and clinical intervention. In doing so, we want to nudge a framework for understanding AWM as a trans-diagnostic mechanism with implications for both diagnosis and cognitive-affective treatment models in social anxiety.

## 2 Method

This review was conducted as a narrative synthesis aimed at integrating and interpreting converging evidence on AWM in social anxiety. Literature was sourced primarily from PubMed, Scopus, and Google Scholar, which together provided broad coverage of biomedical, psychological, and interdisciplinary research. Articles were identified using combinations of keywords (e.g., social anxiety, affective working memory, executive functions) linked by Boolean operators (AND, OR). Given the conceptual heterogeneity of the literature, the search strategy was refined iteratively to capture the most relevant and recent studies rather than relying on a fixed search string.

The final search was conducted on June 15, 2025, and was limited to peer-reviewed English-language human studies.

**Fig 1:**
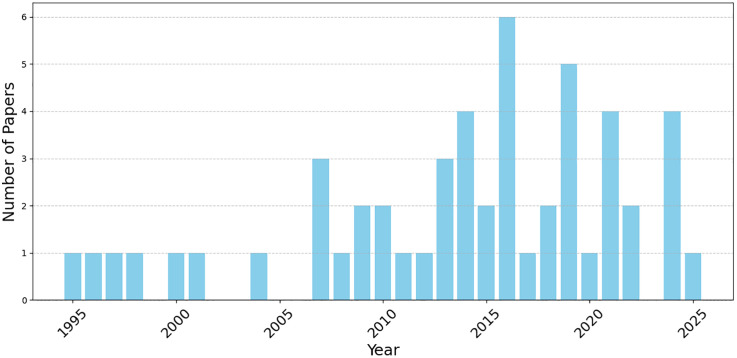
Year-wise distribution of the included articles on AWM and SAD from 1995 to 2025.

The initial search yielded approximately 150 records; after title and abstract screening, 70 articles were examined in full, and 52 were included in the final synthesis. Fig 1 shows the year-wise frequency distribution of the included papers. Screening was conducted by the first author and independently verified by a co-author. Additional studies were identified through backward and forward citation tracing and targeted searches in Google Scholar to include influential pre-prints.

### 2.1 Overview of the review

This narrative review is structured to offer a comprehensive exploration of AWM in the context of SAD, spanning behavioral, electrophysiological, and neuroimaging domains. Section 3 lays the conceptual groundwork, beginning with theoretical models of SAD and executive functioning, followed by an elaboration on affective working memory as a cognitive-emotional interface. Section 4 surveys empirical findings organized thematically across behavioral tasks, neuroimaging paradigms, and ERP based studies, highlighting when and how SAD disrupts AWM processes. Section 5 contains the moderating factors for such impairment. Section 6 re-examines the cognitive theories of SAD against the empirical data. Section 7 addresses major methodological limitations and heterogeneity in the field, drawing attention to inconsistencies in task design, stimuli, and symptom stratification. The final section, section 8, proposes a cohesive agenda for future research, underscoring the clinical utility of AWM-sensitive assessments and pointing to transdiagnostic extensions beyond SAD.

## 3 Conceptual foundations

### 3.1 Theoretical models of social anxiety and cognitive dysfunction

A comprehensive understanding of how social anxiety interacts with AWM requires a theoretical foundation that accounts for both cognitive and affective mechanisms. Several models offer insights into the cognitive-affective disruptions observed in SAD. One of the first influential accounts of the cognitive basis of social anxiety is proposed by Clark and Wells [34]. This model posits that individuals with SAD shift attention inward during social interactions, engaging in excessive self-focused attention (SFA) and forming distorted internal representations of how they believe they appear to others. Following social situations, they often engage in repetitive and negatively biased post-event processing (PEP), which consolidates perceived failures and amplifies anticipatory anxiety. These cognitive processes are inherently resource-demanding and compete for limited-capacity executive functions. As such, they likely disrupt the functioning of WM, particularly in affectively salient contexts involving social evaluation or threat. By co-opting WM resources, SFA and PEP may impair the individual’s ability to encode, maintain, or manipulate task-relevant information, contributing to broader cognitive inefficiencies observed in SAD.

However, building on this intra-cognitive perspective, Rapee and Heimberg’s model [35,36] offers a broader account by integrating both internal and external attentional processes in the maintenance of SAD. In their model, socially anxious individuals generate a negatively biased mental representation of how they are perceived by others, which becomes the basis for real-time social performance monitoring.

Simultaneously, they engage in heightened surveillance of the social environment for cues of negative evaluation, such as frowns, disinterest, or silence. These are often interpreted in line with pre-existing fears. This dual focus on internal states and external social threat results in a hypervigilant, resource-draining attentional loop. Such a loop places substantial demands on WM, particularly in emotionally salient or evaluative contexts, reducing the availability of cognitive resources for goal-directed processing. By framing SAD as a dynamic interaction between cognitive schemas and perceived audience feedback, this model helps explain how attentional control and affective WM processes may become chronically dysregulated in socially anxious individuals.

Adding to the theoretical foundation, a neurocognitive account of how trait anxiety disrupts executive functions such as inhibition and attentional shifting is provided by Attentional Control Theory (ACT) [30]. It suggests that anxiety impairs the goal-directed (top-down) attentional system while enhancing the stimulus-driven

(bottom-up) system. This imbalance compromises the efficiency and effectiveness of WM operations, particularly in emotionally salient contexts. Liang [37] further specified that the attentional control deficit in SAD is especially pronounced in inhibitory functioning, which increases susceptibility to distraction by socially threatening cues.

The Vigilance-Avoidance hypothesis [38,39] adds a temporal dimension, proposing that socially anxious individuals first exhibit hypervigilance toward social threat (early attentional bias), followed by strategic attentional avoidance (late-stage disengagement). Judah e t al. [39] suggests that this disengagement is modulated by WM load, where low WM demands allow avoidance strategies, whereas high load impairs disengagement, leading to sustained attentional capture by threat.

Collectively, these models [30,34,35,38] converge on the notion that SAD is marked by an over-engagement with threat-related information and a concomitant under-engagement of goal-directed attentional processes. These dynamics directly implicate AWM, especially under conditions of high emotional salience or cognitive demand, as explored in subsequent sections.

### 3.2 Affective working memory

WM, as already discussed, is a limited-capacity cognitive system responsible for the temporary maintenance and manipulation of information to be used for goal-directed behavior [25]. Traditional models conceptualize WM as modality-specific storage buffers (e.g., phonological loop, visuospatial sketchpad) governed by a central executive [40]. In this architecture, while maintenance, the temporary holding of information, is a fundamental component of WM; the core executive functions refer to control-related processes that operate on WM contents, such as updating (monitoring and replacing no-longer-relevant information), shifting (switching between tasks), and inhibition (suppressing irrelevant or prepotent responses) [41]. More recent account recognize that WM does not operate in affective isolation, instead, AWM refers to a domain-specific working memory subsystem specialized for maintaining and manipulating emotional feeling states in mind [28]. AWM enables individuals to hold in mind emotions after the emotion-eliciting stimulus is no longer present, thereby supporting goal-directed behavior and complex interactions between emotion and cognition [42]. In this framework, emotions themselves constitute mental representations that can be maintained, compared, and transformed within WM, much like verbal or visuospatial information [28]. Thus, AWM integrates the temporary maintenance of affective experience with executive processes such as updating, inhibition, and shifting, allowing emotional information to guide reasoning, decision-making, and self-regulation AWM differs from neutral WM in two principal ways. First, emotional stimuli, such as socially threatening faces or words in this context, tend to gain prioritized access to WM due to their motivational relevance [43]. This results in automatic encoding and prolonged maintenance of emotionally salient content [28,44], which may displace or interfere with task-relevant, neutral material. Second, affective states modulate executive control processes [45]. For example, anxiety may impair updating or inhibitory control, particularly when task-irrelevant threat distractors are present.

Thus, AWM serves as a critical interface between cognition and emotion, particularly in SAD where threat-related biases, attentional dysregulation, and inhibitory deficits converge to undermine goal-directed WM performance. Hence, we will go into empirical findings that test these interactions directly. For clarity we operationalize terms used throughout the review. **Capacity** refers to the quantitative limit on how much information can be simultaneously stored in WM (e.g., change-detection tasks yielding K values or simple span limits). **Maintenance** refers to the process of keeping a representation active across a retention interval (e.g., delay-period maintenance in a span or change-detection task). **Processing** or **executive** components (updating, manipulation, shifting, inhibition) refer to operations performed on WM contents (e.g., OSPAN combines storage with processing; n-back indexes updating). We will use these operational distinctions below when interpreting task-specific results.

## 4 Empirical evidence on AWM in social anxiety

### 4.1 Working memory function in SAD: maintenance, updating, shifting, and inhibition

A foundational question in understanding AWM in SAD is whether individuals with SAD exhibit generalized impairments in WM, or whether these deficits are content-specific (what information is being processed) and process-dependent (maintenance, updating, and shifting). The following sections reviews empirical findings that directly test these process-specific effects in SAD under different contexts, the summary of which has been provided in Table 1.

**Table 1:** Summary of empirical studies on AWM and executive function in social anxiety, categorized by cognitive domain. SAD = Social Anxiety Disorder; SA = Social Anxiety; HC = Healthy Controls; GAD = Generalized Anxiety Disorder. Studies are grouped by whether the sample involved a *clinical diagnosis of SAD* or *non-clinical/ high-trait SA* participants.

Study (Author, Year)	Sample Size	Sample	Task/ Paradigm
**Capacity/ Maintenance** Amir & Bomyea (2011) [46]	36 SAD, 36 Ctrl.	Clinical SAD	Operation Span (OSPAN) task using threat vs neutral words
Waechter et al. (2018) [47]	25 SAD, 24 Anx. Ctrl., 27 HC	Clinical SAD and anxious controls	OSPAN with social and general threat categories
Moriya & Sugiura (2012) [48]	Expt. 1: 50 (high vs. low SA); Expt. 2: 41 (high vs. low SA); Expt. 3: 33 (high vs. low SA)	Non-clinical	Three visual change-detection paradigms
Yeung & Fernandes (2019) [32]	40 high SA, 40 low SA (×2 experiments)	Non-clinical	Digit span and word span tasks with neutral, general threat, and social threat stimuli
Updating Segal, Kessler & Anholt (2015) [49]	31 high SA, 34 low SA	Non-clinical	Emotional 2-back (facial expressions) assessing updating under affective load
Yoon & Joormann (2017) [33]	31 SAD, 20 HC	Clinical SAD	WM manipulation task with emotional vs. neutral pictures
Yuan & Sun (2024) [50]	29 high SA, 28 low SA	Non-clinical	Emotional word-based N-back with ERP measures
**Shifting** Holder & Prasad (2021) [51]	42 high SA, 42 low SA	Non-clinical	Emotional Wisconsin Card Sorting Task (WCST) measuring cognitive flexibility
**Inhibition** Amir & McNally (1996) [52]	14 SAD, 14 Ctrl.	Clinical SAD	Emotional Stroop task (color-naming of emotional vs. neutral words)
Becker et al. (2001) [53]	32 GAD, 29 SP, 31 HC	Clinical SAD and GAD	EmotionalStroop(word-basedinterference paradigm)
**ERP/ Neuro-imaging** Yuan et al. (2020) [54]	17 SAD, 17 Ctrl	Non-clinical	ERP during visual change-detection (neutral stimuli)
Liang (2024) [55]	35 high SA, 35 low SA	Non-clinical	Change-detection task combined with eye-tracking
MacNamara & Phan (2019) [31]	76 (trans-diagnostic clinical sample)	Clinical	WM task with interspersed neutral and negative images; ERP and behavioral data
Geiger & Domschke (2016) [17]	15 SAD, 15 Ctrl.	Clinical SAD	Resting-state fMRI of executive control network connectivity
Liao & Qiu (2010) [56]	22 SAD, 21 Ctrl.	Clinical SAD	Resting-state fMRI of amygdala connectivity

#### 4.1.1 Capacity and maintenance.

Amir and Bomyea [46] examined “working memory capacity” (WMC) in individuals with generalized social phobia using an Operation Span (OSPAN) task that combines word recall with interleaved arithmetic judgments. Although they have used the term WMC measure, this paradigm primarily indexes maintenance under concurrent executive-attention demands [57]. The authors found that participants with social anxiety performed worse than controls for neutral words but not for socially threatening words, indicating that when threat information is task-relevant,

executive-attention–based maintenance may be preserved or even facilitated. This can be interpreted as evidence for preferential processing of threat-congruent stimuli in WM. However, the main concern with the study was that trait and state general anxiety and depression were all high in the sampled SAD participants. So, we can’t pinpoint the deficit to SAD. Additionally, there was no control condition using general threat words alongside social threat words. As a result, it is not possible to determine whether the observed differences in WM performance are attributable specifically to the social nature of the stimuli or merely to any threatening content.

A direct replication attempt by Waechter et al. [47] taking care of these limitations by introducing an anxiety control group and general threatening words along side social threatening words failed to reproduce these findings: across SAD, anxious control, and healthy control groups, there were no significant differences in WM performance for either social threat, general threat, or neutral stimuli. This inconsistency raises critical concerns about methodological differences and diagnostic heterogeneity.

Yoon et al. [33] found no differences between an SAD group and non-anxious controls in the ability to maintain information in WM for emotional content. However, when it comes to the ability to manipulate information in WM (recall the list of stimuli in backward order), individuals with SAD demonstrated better manipulation (i.e., sorting) of emotional versus neutral material in WM tasks. While healthy controls showed increased difficulty with emotional content, SAD participants exhibited reduced sorting costs for emotional stimuli, especially negative ones. This suggests that the affective valence of WM content can differentially modulate cognitive control depending on anxiety status.

Moriya and Sugiura [48] assessed visual working memory capacity using a change detection paradigm that estimates the number of items (K) an individual can retain simultaneously without concurrent processing. This task measures pure storage capacity, distinct from executive maintenance or manipulation. Their results indicated that individuals with higher trait social anxiety showed increased visual storage capacity when no distractors were present but exhibited impaired filtering efficiency under distracting conditions, suggesting that anxiety-related attentional control deficits may offset otherwise high capacity.

Although at first glance, the findings of Amir and Bomyea [46] and Moriya and Sugiura [48] may appear contradictory—given that both refer to “working memory capacity” yet report opposing directions of effect—the difference lies primarily in the operational definition of WMC and the cognitive demands of the paradigms used. Amir and Bomyea’s complex-span task indexes maintenance under concurrent executive-attention load, whereas Moriya and Sugiura’s change-detection task captures pure storage capacity without processing interference. Thus, rather than conflicting, the two studies jointly illustrate that individuals with social anxiety can exhibit intact or even enhanced storage capacity for affectively salient information, while showing reduced efficiency when executive control or attentional regulation is taxed.

Further clarity on the effect of content specificity on AWM in social anxiety comes from Yeung and Fernandes [32], who demonstrated altered WM capacity in individuals with high social anxiety specifically in response to socially threatening words, but not to general threat or neutral words. Critically, they controlled for semantic similarity within word lists. This ensured that the impairment in WM was not due to increased semantic clustering or associative interference. They also pointed the null findings in Waechter [47] could be due to the arithmetic task being performed as a part of the complex span task. Thus, they chose a simple span task [58] for the study providing compelling evidence that the meaning (specifically the socially evaluative nature of the words), rather than lexical overlap or associative interference, was the driving factor behind the observed reduction in WM capacity.

Overall, findings on WM in social anxiety suggest a context and affect-dependent profile. While some studies [33,47] indicate preserved or enhanced WM for socially threatening content, especially when emotionally relevant, others [48,59] report impairments under high load or distraction, likely reflecting deficits in top-down control.

Notably, some studies could conflate pure storage capacity (e.g., change-detection K values, simple span maxima) with maintenance under load (e.g., complex span tasks like OSPAN) [46]. For interpretive clarity, we recommend distinguishing whether an effect reflects: (a) reduced storage capacity (fewer items representable), (b) impaired maintenance under concurrent processing (difficulty keeping items active while performing another task).

#### 4.1.2 Updating.

WM updating is the continuous monitoring, substitution, and maintenance of task-relevant information. In socially anxious individuals, this core executive process appears selectively impaired in contexts involving affective content.

Segal et al. [49] employed an 2-back task (a task typically used for assessing WM updating) [60] with affective stimuli and found found that individuals with high social anxiety were less affected by distracting positive stimuli, indicating greater suppression of positive emotional content. This pattern suggests an active cognitive bias against integrating affirming stimuli into WM, possibly contributing to the maintenance of negative self-beliefs.

Extending this, Yuan et al. [50] assessed updating performance using an emotional word-based 2-back paradigm. Results revealed that high socially anxious participants responded more slowly to positive words than to negative or neutral ones, whereas low-anxiety individuals showed no such discrepancy. Interestingly, these reaction time

(RT) effects were not accompanied by differences in accuracy, aligning with Attentional Control Theory’s [30] prediction that anxiety more severely impacts processing efficiency than performance effectiveness. The study interpreted these findings as reflecting a retrieval-specific deficit in updating positive content, reinforcing the notion of an under-processing of positive social feedback in socially anxious individuals.

Complementing these findings, Liang [55] used eye-tracking while performing a n-back task in which emotional face pairs were used as distractors. The study demonstrated that task-irrelevant angry (threatening) faces selectively impaired WM updating efficiency (measured by RT) in socially anxious participants, but not in non-anxious controls. Eye-tracking revealed that socially anxious individuals showed increased initial fixations and longer dwell times on angry faces, indicating an early attentional capture and sustained engagement with threat stimuli. Crucially, these attentional biases interfered with the updating process, but only under higher cognitive load (2-back versus 0-back), emphasizing the interplay between emotional distractibility and executive capacity.

While affective working memory tasks focus on the online manipulation of emotional information, recent work also implicates broader updating inflexibilities in social anxiety. For instance, Zabag et al [61] demonstrated that individuals with high social anxiety exhibit a specific deficit in positive belief updating during social learning tasks, even when counterfactual feedback explicitly reveals that avoidance is costly. Although this paradigm does not directly tap working memory processes, it converges with AWM research in highlighting a generalized rigidity in integrating new affective evidence, particularly positive social information. This pattern supports the view that impaired affective updating may extend beyond transient WM operations to more enduring belief-updating mechanisms.

Together, these findings [49,50,55] reveal that WM updating impairments in social anxiety are affectively biased and context-sensitive, with deficits emerging particularly for positive stimuli (under-integration) and threatening distractors (over-attention).

#### 4.1.3 Shifting and cognitive flexibility.

Shifting is the ability to switch between different tasks or ways of thinking [41]. This skill plays an important role in SAD, where people often struggle to adjust their thoughts in stressful situations. Research using tasks like the Wisconsin Card Sorting Test (WCST) [62], which measures this kind of mental adaptability, supports this idea. In one study [51], individuals with high levels of social anxiety made more repeated mistakes, called perseverative errors, when the rules of the task changed. These same individuals also had more difficulty coming up with helpful, alternative ways of thinking during a therapeutic exercise called cognitive restructuring. This suggests that when people with social anxiety are emotionally distressed, their ability to shift may be impaired, making it harder for them to reframe anxious thoughts.

#### 4.1.4 Inhibition.

There are studies on inhibitory control in the context of social anxiety and WM, but they are relatively fewer and often overlap conceptually with attentional control or are embedded within broader executive functioning paradigms rather than being tested as pure inhibition tasks. For example, the emotional Stroop task which measures delayed color-naming of emotionally salient words, has shown that socially anxious individuals exhibit greater interference from socially threatening words, suggesting difficulty inhibiting the processing of emotional distractors [52,53]. Similarly, the emotional Go/No-Go task that requires participants to withhold responses to specific emotional stimuli has also revealed that anxious individuals are more prone to false alarms when no-go cues involve threatening faces [63]. In WM paradigms, inhibitory control is rarely tested in isolation but is often inferred from impaired performance in tasks involving emotional distraction, such as slower reaction times or reduced accuracy on emotional n-back tasks [55]. These findings suggest that inhibition may contribute to AWM disruptions in SAD, particularly under conditions of high emotional salience, though its influence is frequently intertwined with updating and attentional disengagement.

Taken together, the behavioral literature explained in this section, paints a picture of WM functioning in SAD that is both specific and dynamic. Basic WM maintenance appears largely intact under neutral conditions, a finding that has been replicated across span and updating tasks. However, when WM tasks include emotionally salient, socially evaluative, or personally relevant content, individuals with SAD consistently show performance disruptions. Critically, deficits in updating, shifting, and inhibitory control are not universal across WM tasks but appear conditional on affective load, cognitive demand, and stimulus relevance. Socially anxious individuals do not simply forget more, they forget differently: they are slower to disengage from negative content, resistant to integrating positive cues, and cognitively inflexible when required to shift or inhibit attention. These process-specific disruptions suggest that AWM impairment in SAD reflects not a failure of capacity but a failure of regulation, emerging at the intersection of affective salience and executive strain.

### 4.2 Electrophysiological and neuroimaging correlates

Advances in cognitive neuroscience have enabled the identification of neural mechanisms underlying AWM impairments in SAD. Two electrophysiological markers, contralateral delay activity (CDA) and the late positive potential (LPP), have emerged as particularly informative, alongside functional neuroimaging studies highlighting disrupted fronto-limbic dynamics.

CDA reflects the number of items actively held in visual WM and is typically measured over posterior parietal electrodes. In an ERP study by Yuan et al. [54], individuals with high social anxiety exhibited significantly larger CDA amplitudes than low-anxiety controls when maintaining four neutral visual items in a change-detection task. This suggests that high socially anxious individuals may recruit greater WM resources even for non-affective content, likely as a compensatory mechanism. However, behavioral performance (i.e., K value accuracy) did not differ between groups, highlighting a dissociation between efficiency and effectiveness which is consistent with ACT [30].

In contrast, the LPP, which is a centro-parietal ERP component sensitive to emotional salience, reveals how affective stimuli modulate attention and WM processes. MacNamara et al. [31] found that individuals high in social anxiety or panic symptoms showed increased LPP amplitudes in response to negative images, particularly under low working memory load. This implies that when executive control is not fully taxed, emotional distractors gain prioritized access to working memory stores. Conversely, under high cognitive load, socially anxious individuals failed to suppress these affective responses effectively, indicating diminished top-down control under resource constraints.

Neuroimaging studies corroborate and extend these findings. Geiger et al. [17], using resting-state fMRI and independent component analysis, demonstrated altered functional connectivity within the executive control network in SAD. Specifically, they observed reduced orbitofrontal connectivity and increased coupling between the orbitofrontal cortex and the amygdala. These are key nodes in emotion regulation and salience detection. This pattern suggests a shift toward bottom-up threat reactivity and impaired prefrontal modulation of limbic activity.

Moreover, Liao et al. [56] reported that elevated social anxiety was associated with hyperactivation of the amygdala and blunted modulation by cognitive load during emotional picture processing, further emphasizing the inflexibility of affective attention systems in SAD. These alterations in neural circuitry are consistent with reduced inhibitory control and sustained engagement with emotionally salient distractors, key features of AWM disruption in social anxiety.

Taken together, electrophysiological [31,54] and neuroimaging [17,56] evidence converge to highlight a neural architecture in SAD characterized by heightened affective salience detection, increased resource allocation to non-task-relevant stimuli, and compromised top-down control from prefrontal systems. These neurocognitive alterations are especially pronounced when WM is taxed by emotional or socially threatening content, aligning with behavioral findings of selective AWM dysfunction in SAD.

## 5 Moderating and mediating factors

While AWM impairments in SAD are well-documented, their manifestation appears to be moderated by a range of cognitive, contextual, and individual difference variables. Accumulated literature of WM and SAD points to two such influences that could benefit from further discussion: the impact of working memory load and the role of symptom dimensions and comorbidity.

### 5.1 Working memory load

WM load plays a crucial moderating role in the interaction between cognitive control [64]. The extent to which emotionally salient stimuli impair WM performance is not static but varies as a function of task demands [65]. This relationship provides a powerful lens for understanding how executive capacity constraints exacerbate affective biases in SAD.

Under low WM load, individuals with SAD may successfully engage in avoidance strategies to minimize processing of threatening stimuli. For instance, Judah et al. [39] demonstrated that high socially anxious participants exhibited attentional avoidance of disgust faces (a socially threatening stimulus) when performing a dot-probe task, which checks how easily someone’s attention is drawn to and/or away from specific stimuli, under low cognitive load. This behavior aligns with the “vigilance-avoidance” model, which suggests an early attentional bias toward threat, followed by strategic withdrawal.

However, this avoidance pattern diminishes or reverses under high cognitive load. In the same study [39], participants with high social anxiety exhibited difficulty disengaging from socially threatening faces when concurrently performing a high-load n-back task. These results indicate that the cognitive resources required for attentional control are compromised when the WM system is taxed, thereby exposing individuals to prolonged engagement with threat. The implication is that WM load interferes with the ability to inhibit or redirect attention, thereby amplifying the influence of affective distractors.

Adding to this, MacNamara et al. [31] found that WM load plays a key role in emotional processing. When participants were asked to remember fewer items (low WM load), individuals with higher social anxiety showed stronger brain responses, as evident by larger LPPs to negative images. However, when the task became more demanding (high WM load), this heightened emotional response diminished. While this might seem like improved emotional regulation under pressure, the researchers suggest it may actually reflect a “resource depletion” effect, meaning the brain is so occupied with the difficult task that it no longer has the capacity to fully react to emotional distractions. This finding suggests that executive resources serve as a buffer against affective interference, when available, they support disengagement and goal-maintenance; when depleted, they fail to suppress emotionally salient intrusions.

### 5.2 Symptom dimensions and comorbidity

SAD rarely presents in isolation; rather, it frequently co-occurs with other internalizing disorders [64] such as generalized anxiety disorder (GAD) [66], panic disorder (PD), and depression [67]. These comorbidities introduce notable heterogeneity [68] in both emotional reactivity and executive functioning, making it challenging to isolate AWM-specific deficits attributable solely to SAD. Therefore, understanding the cognitive-affective processes underpinning SAD requires considering comorbid symptom dimensions, as they may independently or interactively modulate attentional control, emotional processing, and WM performance.

A growing body of work has adopted a dimensional and transdiagnostic perspective to elucidate these interactions. Notably, MacNamara et al. [31] examined AWM in a clinically heterogeneous sample. Several studies indicate distinct comorbidity-specific signatures on AWM. For example, work on generalized anxiety and panic-related samples links these disorders to prefrontal dysfunction and reduced efficiency in executive control, leading to broader impairments in updating and inhibition under load (see [69,70]). By contrast, major depressive disorder has been associated with reduced updating for positive material and reward-insensitivity, which would specifically hinder the incorporation of positive social feedback into WM [71,72]. MacNamara et al. [31] further show that panic and depressive symptom dimensions modulate LPP and WM load effects differently, underscoring that comorbid symptom clusters can push AWM dysfunction in different directions (e.g., avoidance vs. executive overload). Hence, adopting RDoC-aligned approaches [73], which encourages moving beyond fixed diagnostic categories and instead studying core psychological processes, like vigilance, avoidance, and cognitive control, across different types of internalizing symptoms.

Despite these advances, there remains a paucity of research directly investigating how comorbid conditions influence AWM in SAD. Most existing studies either do not explicitly model comorbidity or lack the statistical power to disentangle its specific contributions. Given the high prevalence and clinical relevance of comorbidity in SAD [74], future studies should take a dimensional approach. For example, instead of focusing only on diagnostic categories, researchers could examine how varying levels of social anxiety, low positive mood, or panic symptoms relate to AWM performance. This would make it possible to understand how specific symptom traits influence their AWM, regardless of whether someone meets criteria for a particular disorder.

In parallel, targeted group comparisons can help clarify the role of particular comorbidities when they are especially relevant to a given cognitive domain. For instance, because major depressive disorder (MDD) is strongly linked to deficits in motivational disengagement [75,76], one might expect MDD to particularly affect AWM under especially effortful task conditions. In such cases, directly comparing individuals with pure MDD, pure SAD, and comorbid SAD–MDD in their ability to manipulate affective versus neutral content in WM would offer valuable insights.

## 6 Rethinking AWM disruption in SAD

Empirical findings on AWM in SAD strongly support the idea that affective salience disrupts executive functioning [39], particularly under high cognitive load [31] or emotional self-relevance [77]. This pattern aligns well with ACT, which posits a load-sensitive breakdown in top-down regulation. Yet, ACT assumes a relatively uniform impairment in goal-directed attention under anxiety, while studies like Judah et al. [39] and Yeung and Fernandes [32] show that SAD-related impairments are not purely capacity-based but exhibit content specificity. This can be better explained by models that take into account how individuals personally evaluate the emotional meaning of a stimulus (stimulus appraisal) and how strongly that stimulus captures their attention because it feels important or self-relevant (motivational salience).

**Fig 2:**
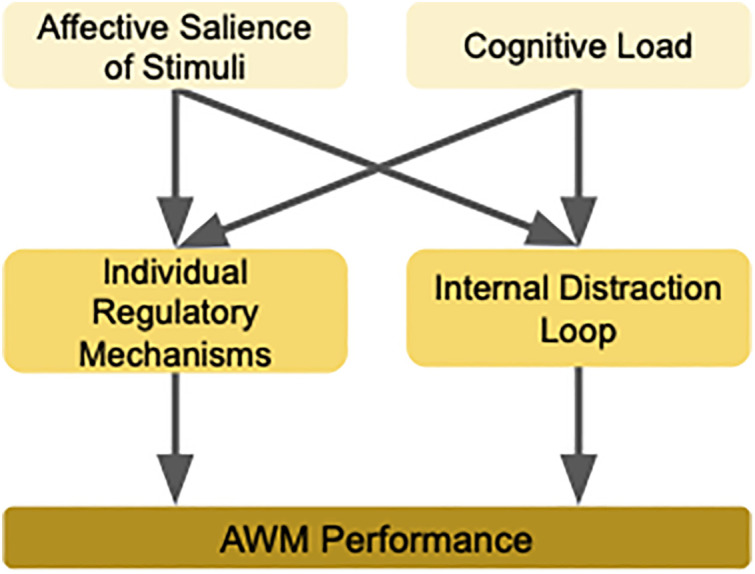
Affective Working Memory Disruption in SAD: The schematic illustrates four interacting mechanisms hypothesized to underlie impairments in affective working memory (AWM) among socially anxious individuals: (1) affective salience of stimuli, which increases vulnerability to emotional distractors; (2) cognitive load, which exacerbates regulatory failures under executive strain; (3) individual regulatory mechanisms, such as attentional control, trait mindfulness, fear of positive evaluation and cognitive flexibility; and (4) internal distraction loops, including rumination and anticipatory anxiety. These factors jointly contribute to dysregulated working memory processing in social anxiety disorder, particularly in emotionally salient contexts.

All the empirical evidence (outlined in section 4) suggests that impairments emerge not from a general deficit in capacity or attention, but from the interaction of four key mechanisms as depicted in Fig 2 as well:

1 **Affective Salience of Stimuli:** Socially anxious individuals show disproportionate interference from stimuli that are not just negative, but those stimuli that are emotionally salient or attention-grabbing to specific individuals, based on their personal experiences, fears, values, or social schemas.
2 **Cognitive Load:** Deficits in AWM become pronounced under high cognitive load, consistent with resource depletion theories, but may be masked under low-load conditions.
3 **Individual Regulatory Mechanisms:** There can exist inter-individual variability in terms of traits like attentional control, cognitive flexibility, emotion regulation style, or even trait mindfulness, and this in turn modulates how AWM is impacted. Additionally, fear of positive evaluation (FPE), the apprehension that positive feedback will heighten social scrutiny, may further bias updating by dampening the encoding and integration of positive social information within AWM.
4 **Internal Distraction Loop:** Internal factors like rumination and anticipatory anxiety compete with task demands, functioning as “covert distractors” that drain WM resources even in the absence of external threat.

## 7 Methodological considerations and gaps

The AWM and SAD literature remains constrained by several methodological limitations that complicate their interpretation and limit generalizability. Identifying and addressing these gaps is essential for the refinement of theoretical models and the development of effective interventions.

First, considerable heterogeneity exists in task paradigms used to assess AWM. Studies [46,48,78], for example, checking capacity only, can employ a variety of WM tasks, including the OSPAN [46], digit/word span [78], and change-detection paradigms [48]. These tasks differ in the specific cognitive demands they place on the participant, with some emphasizing storage capacity (e.g., simple span tasks), others engaging both storage and processing (e.g., OSPAN), and still others focusing on visual short-term memory precision (e.g., change-detection). Thus, they may each tap into distinct sub-components relevant to affective processing. Without standardized tasks or cross-paradigm comparisons, it is difficult to disentangle task-specific effects from broader AWM impairments.

Second, the emotional stimuli used across studies vary widely in modality (e.g., words [50], faces [49], pictures [33]), valence (e.g., general threat [33], social threat [78]), and relevance to the individual. Since, there is such variety in stimulus type, one can systematically examine how different stimulus types of emotional content influence particular sub-processes of working memory, specifically for SAD participants. Without such delineation, isn’t it difficult to assess whether findings derived from one class of stimuli (e.g., socially threatening words) can be meaningfully generalized to others (e.g., aversive images or faces)?

Third, we couldn’t find studies that employ longitudinal or ecologically valid designs.

Almost all of the findings derive from single-session lab tasks, which may not fully capture the context-sensitive nature of AWM in daily social functioning. The interaction between chronic affective states, momentary anxiety, and WM performance remains poorly understood. Ambulatory assessment or real-time AWM tracking in naturalistic environments could provide deeper insight into how affective interference unfolds outside of laboratory conditions. Here, the increasing ubiquity and sensor-rich capabilities of smartphones offer a promising solution. By leveraging smartphone-based experience sampling methods (ESM) [79,80], researchers can collect real-time data on emotional states, cognitive load, and contextual factors multiple times throughout the day. Additionally, smartphones can administer brief cognitive tasks adapted from AWM paradigms outside of the laboratory, allowing actively repeated measurements in naturalistic settings. Built-in sensors (e.g., GPS, accelerometer, screen use, app activity) can passively track behavioral indicators relevant to social engagement and stress exposure. This combination of active and passive data collection can enable a detailed, temporally dynamic understanding of how affective interference impacts AWM during real-world social interactions. Ultimately, such approaches could clarify the ecological validity of lab-based findings and illuminate how fluctuations in anxiety influence cognitive control in everyday life.

Fourth, in SAD and AWM studies, there is limited attention to individual difference variables such as individual cognitive capacity, trait mindfulness, or emotion regulation strategies. These baseline cognitive capacities/factors can act as moderators, meaning they influence the strength or direction of the relationship between anxiety and AWM performance. Incorporating moderator variables would improve predictive specificity and allow for more refined, individualized models of how anxiety interacts with AWM.

Fifth, as discussed in previous section (section 5) comorbidities are often insufficiently controlled. Although some studies have accounted for depression or general anxiety symptoms [31,47], many still rely on binary SAD diagnoses without controlling for overlapping symptom dimensions. This limits the ability to attribute AWM effects specifically to social anxiety, rather than to general distress or neuroticism.

In sum, the current literature is characterized by methodological fragmentation, stimulus inconsistency, and a lack of translational or longitudinal approaches. These methodological inconsistencies are compounded by pervasive statistical power limitations in affective and cognitive neuroscience. As Button et al. [81] demonstrated, the median power of published neuroscience studies is often below 25%, leading to inflated effect sizes, excess significance, and low reproducibility. This concern is particularly salient for AWM studies, which frequently rely on small samples and complex designs. Consequently, some apparent discrepancies in the literature may reflect underpowered designs rather than true theoretical divergence. Future AWM research in social anxiety would benefit from preregistered, adequately powered studies, ideally coupled with open data practices to enhance reliability and cumulative progress.

## 8 Clinical and translational implications

Naturally, there should be clinical relevance for all the above mentioned findings that hint towards AWM impairment in SAD. This section discusses how it can have translational implications for clinical assessment, intervention design, and mechanistic models of treatment response.

One major avenue is the development of targeted cognitive training interventions. Both working memory training (WMT) [82] and attention bias modification (ABM) [83] have shown promise in enhancing top-down control over socio-affective interference. A novel direction would be to integrate these approaches, for instance, embedding ABM within emotionally loaded WM span tasks that simulate real-world social demands (e.g., responding to feedback while holding self-relevant goals in mind). These dual-target designs could directly remediate both attentional and WM dysfunctions underlying SAD.

Current psychotherapies, particularly CBT [84], could be enhanced by explicitly integrating AWM demands into exposure exercises, e.g., having patients maintain a prosocial goal (e.g., making eye contact) while simultaneously processing evaluative cues. Likewise, mindfulness-based interventions may indirectly bolster AWM by reducing rumination, which depletes WM resources and sustains anxious preoccupation.

A novel therapeutic target is AWM updating biases, particularly difficulty incorporating positive feedback into self-concept. These deficits may hinder corrective learning in social contexts, leading to persistence of negative self-evaluation. Interventions that focus on structured reflection on feedback during post-event processing may improve AWM flexibility and reduce avoidance.

In sum, translational applications of AWM research hold strong promise for improving assessment precision, developing mechanism-based interventions, and enhancing personalization across the clinical trajectory of social anxiety.

## 9 Conclusion

This narrative review highlights AWM as a critical intersection of cognition and emotion in SAD. While basic WM capacity may remain intact in neutral contexts, impairments emerge reliably when socially salient or emotionally threatening stimuli are introduced. These disruptions are particularly evident in tasks requiring updating, shifting, and inhibition, executive functions taxed by anxiety-driven cognitive biases.

Electrophysiological and neuroimaging studies corroborate behavioral findings, showing hyperactivation of salience-detection circuits (e.g., amygdala) and compromised prefrontal regulation. Moderating factors such as cognitive load, stimulus relevance, and comorbid symptoms further nuance the manifestation of AWM dysfunction. However, the field faces methodological challenges, including task heterogeneity, limited ecological validity, and insufficient control of internal distractors like rumination. Clinically, AWM offers a promising target for cognitive training and precision assessment. Ultimately, integrating AWM into transdiagnostic frameworks may sharpen our mechanistic understanding of SAD and lead to more tailored, effective interventions.
